# Efficacy of voice training on benign vocal cord lesions after surgery: A systematic review, meta-analysis, and trial sequential analysis of randomized clinical trials

**DOI:** 10.1097/MD.0000000000044024

**Published:** 2025-08-29

**Authors:** Xiaolan Zhang, Chaobing Liu, Zhitao Fan, Zhenhua Qiao, Man Liu

**Affiliations:** a Department of Otolaryngology, Hebei Eye Hospital, Xingtal City, Hebei Province, China.

**Keywords:** benign vocal cord lesions, meta-analysis, surgery, voice therapy, voice training

## Abstract

**Background:**

Voice training (VT) aids patients in achieving or restoring physiological harmony among the vocal organs and in correcting the faulty conditioned reflexes induced by benign vocal cord lesions (BVLs). Herein, we designed a systematic review and meta-analysis for the first time to evaluate the effect of VT on BVLs after surgery based on several outcomes.

**Methods:**

Four electronic databases (PubMed, Web of Science, Scopus, and Cochrane Library) and 3 Chinese databases (CNKI, VIP, and Wanfang) were searched through September 26, 2024, without any restrictions. We used Review Manager, version 5.3 for meta-analysis and presented the data as standardized mean difference (SMD) or risk ratio (RR) and 95% confidence interval (CI).

**Results:**

A total of 1771 records were retrieved from 7 databases that 22 articles were entered into the meta-analysis. The pooled RR for total effective rate was 1.14 (*P* = .0009). The pooled SMD for G was −1.64 (*P* < .00001), for R was −1.76 (*P* < .00001), and for B was −3.70 (*P* < .0001). The results showed a 14% improvement in the intervention group compared to the control group. The pooled SMD for VHI-T was −1.57 (*P* < .00001), for VHI-E was −1.04 (*P* < .00001), for VHI-F was −0.93 (*P* < .00001), and for VHI-P was −1.11 (*P* < .00001). The pooled SMD for jitter was −1.55 (*P* < .00001), for shimmer was −1.29 (*P* < .00001), for maximum phonation time (MPT) was 0.80 (*P* < .00001), for dysphonia severity index (DSI) was 2.63 (*P* < .0001), for NHR was −0.73 (*P* = .05).

**Conclusions:**

The intervention group showed marked improvements across various metrics compared to the control group, with a 14% increase in total effective rate and notable reductions in G, R, B, VHI-T, VHI-E, VHI-F, VHI-P, jitter, and shimmer. There were also significant increases in MPT and DSI, with NHR remaining unchanged.

## 1. Introduction

Benign vocal lesions (BVLs) are non-cancerous growths on the vocal cords. Common types include singer’s nodules, polyps, papillomas, Reinke edema, and cysts. Other types are sulcus vocalis, mucosal bridges, intracordal cysts, vocal cord varices, and anterior webs. These lesions can develop due to factors such as vocal abuse, overuse or misuse of the voice, chronic upper airway infections, allergies, smoking, and gastroesophageal reflux. Frequent coughing and throat-clearing can also irritate the mucosa, worsening the voice.^[1–3]^

Voice disorders cause communication handicap which leads on to psychosocial problems and impaired quality of life.^[4,5]^ Certain benign mucosal lesions are strongly associated with age and especially with gender. These differences may be explained by intrinsic differences in laryngeal anatomy and phonatory physiology in these groups, including differences in phonatory frequency and air pressure, and in the ability of the membranous vocal fold to withstand phonotrauma.^[6]^ Phonomicrosurgery, including the use of CO_2_ laser and cold excision, can improve the voice handicap in patients with BVLs with a low recurrence rate. This confirmed the important role of phonomicrosurgery in the treatment of BVLs.^[7,8]^

Although voice therapy is primarily indicated for treating functional dysphonia without organic abnormalities in the vocal folds, many clinicians have also applied it to dysphonic patients with benign mass lesions on the vocal folds.^[9,10]^ The effectiveness of voice therapy for vocal disturbances associated with BVLs is thought to be due to the regression of lesions and the correction of excessive or inappropriate muscle contractions of the phonatory organs.^[9,11,12]^ Among various voice therapies, voice training (VT) helps patients establish or reestablish physiological balance among the vocal organs and correct the erroneous conditioned reflex caused by BVLs.^[13]^ VT treatment aids in removing the control of the erroneous conditioned reflex, changing vocal habits, and establishing a new conditioned reflex to restore normal phonation function.^[14,15]^

Herein, we designed a systematic review and meta-analysis for the first time to evaluate the effect of VT on BVLs after surgery based on several outcomes.

## 2. Materials and methods

The study followed the guidelines for the preferred reporting items for systematic reviews and meta-analyses (PRISMA) protocol.^[16]^

### 2.1. Literature search

Four electronic databases (PubMed, Web of Science, Scopus, and Cochrane Library) and 3 Chinese databases (CNKI, VIP, and Wanfang) were searched through September 26, 2024, without any restrictions by one author (Z.F.). The search terms were (“vocal fold polyp*” or “vocal polyp*” or “vocal cord polyp*” or “BVL*” or “benign vocal fold lesion*” or “voice disorder*” or “vocal disorder*” or “benign vocal” or “benign voice” or “vocal cord nodule*” or “vocal fold nodule*” or “vocal cord cyst*” or “vocal fold cyst*” or “vocal cord scar*” or “vocal fold scar*” or “vocal cord granuloma*” or “vocal fold granuloma*” or “vocal cord hemorrhage” or “vocal fold hemorrhage”) and (“VT” or “vocal training”). We also went through the references of eligible studies and manually reviewed articles to identify possible relevant publications, as well as through electronic sources such as Google Scholar. It is important to note that ethical approval was not needed as this study involved retrieving and synthesizing data from previously published articles. Another author (X.Z.) rechecked all processes the literature search. The difference was resolved by a short conversation.

### 2.2. Study selection

The PICOS framework was utilized as the inclusion criteria for this study. P (population): patients with BVLs who have undergone surgery. I (intervention): VT programs or therapies administered post-surgery. C (comparison): Standard care or no VT post-surgery. O (outcomes): maximum phonation time (MPT), voice handicap index (VHI) scores (total, physiological, functional, and emotional), Jitter, Shimmer, dysphonia severity index (DSI), noise-harmonic ratio (NHR), and GRB score (G: grade. R: roughness. B: breathiness). S (study design): randomized clinical trials (RCTs).

The criteria for inclusion encompass all RCTs that have an intervention group as a case group receiving including surgery and routine therapy (i.e., routine nursing and conventional treatment) or surgery alone combined to VT, and a control group receiving surgery and routine therapy or surgery alone. The criteria for exclusion consist of studies that involve participants with a history or diagnosis of any systemic diseases that overlap with vocal cord disorders, cases of vocal cord disorders that are undergoing therapies other than Western medicine and for other exclusion criteria, please see the article of Sheikhi et al.^[17]^

### 2.3. Data extraction

Two authors (X.Z. and C.L.) independently conducted a review of the literature and data extraction to maintain consistency in the screening core criteria and data collection process. The differences between them were resolved by third author (Z.F.).

### 2.4. Statistical analysis and data synthesis

We used Review Manager, version 5.3 (RevMan 5.3; the Cochrane Collaboration, the Nordic Cochrane Centre, Copenhagen, Denmark) was used to compute the effect sizes mirroring the standardized mean difference (SMD) along with a 95% confidence for meta-analysis and presented the data as SMD or risk ratio (RR) and 95% confidence interval (CI). The studies’ heterogeneity was evaluated using the *I*^2^ statistic, with the significance level set at *P* < .05.^[18,19]^ Considering the likely heterogeneity of the studies, a random-effects model was applied in the meta-analysis when the *P*_heterogeneity_ was <.10 (*I*^2^ exceeding 50%). If not, a fixed-effect model was utilized.^[20,21]^ The presence of publication bias was assessed using a funnel plot and Begg and Egger tests, with the significance level set at *P* < .10.^[22,23]^ The comprehensive meta-analysis version 2.0 (CMA 2.0; Biostat Inc., Englewood) software was employed to conduct publication bias and sensitivity analyses.

The ‘risk-of-bias tool’ recommended by the Cochrane Handbook for Systematic Reviews of Intervention was used to assess the methodological quality of the included studies. This 2-part tool concentrates on 6 distinct areas: sequence generation, allocation concealment, blinding, incomplete outcome data, selective outcome reporting, and other sources of bias. Each area includes one or more specific entries. The second part of the tool involves making a judgment about the risk of bias for that entry.^[24,25]^ Two authors (Z.Q. and M.L.) independently revised the articles for the risk of bias and the differences were resolved by a short discussion.

A trial sequential analysis (TSA) was performed using TSA software (version 0.9.5.10 beta) (Copenhagen Trial Unit, Centre for Clinical Intervention Research, Rigshospitalet, Copenhagen, Denmark).^[26]^ The necessary information size (RIS) for blood cytokine levels was determined with an alpha risk of 5% and a beta risk of 20%.^[27,28]^ If the Z-curve intersected the RIS, it indicated that the studies had a sufficient number of cases, and the conclusion could be considered reliable.^[29]^

## 3. Results

### 3.1. Study selection

**Figure 1.**
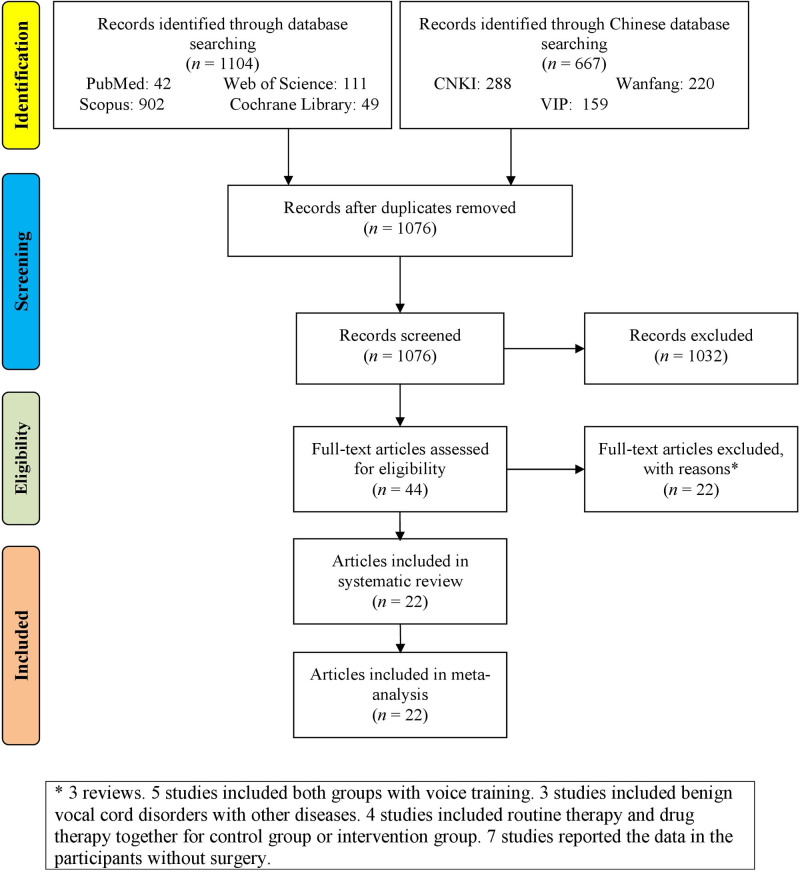
Flowchart of the study selection.

Figure 1 provides a detailed visual representation of the rigorous selection process for studies in a systematic review or meta-analysis. Initially, records are identified through database searching, with 1104 records from PubMed, Web of Science, Cochrane Library, and Scopus databases, and an additional 667 records from Chinese databases (CNKI, Wanfang, and VIP). After removing duplicates, 1076 records remain, which are then screened based on titles and abstracts, resulting in the exclusion of 1032 records. This leaves 44 full-text articles to be assessed for eligibility, of which 22 articles are excluded for various reasons. Ultimately, 22 articles^[13,30–50]^ are included in the qualitative synthesis and then the quantitative synthesis (meta-analysis).

### 3.2. Studies’ characteristics

Table 1 shows the characteristics of various studies included in the meta-analysis. The studies were conducted between 2009 and 2024 involving different durations of VT, ranging from 2 to 12 weeks. Outcome measures include MPT, VHI, Jitter, Shimmer, DSI, NHR, and GRB score.

**Table 1:** Characteristics of the studies included in the meta-analysis.

First author, publication year	Number of interventions/cases	Intervention group	Control group	Duration of voice training, weeks	Outcome measures
Fengqin, 2019^[30]^	30/30	SRT + VT	SRT	4	MPT
Gang, 2009^[31]^	71/71	SRT + VT	SRT	4	Total effective rate
Haixia, 2021^[32]^	41/411	SRT + VT	SRT	2	VHI-T
Huihui, 2024^[33]^	50/30	SRT + VT	SRT	10	VHI-E, VHI-F, VHI-P, G, total effective rate, Jitter, Shimmer
Jiajun, 2015^[34]^	60/60	Surgery + VT	Surgery	4	Total effective rate
Jie, 2018^[35]^	46/50	Surgery + VT	Surgery	6	MPT, Jitter, Shimmer, DSI, NHR
Li, 2022^[36]^	39/39	SRT + VT	SRT	12	MPT, VHI-T, VHI-E, VHI-F, VHI-P, Total effective rate, Jitter, Shimmer
Lin, 2014^[13]^	30/30	SRT + VT	SRT	12	MPT, VHI-T, G, R, B, Jitter, Shimmer, NHR
Lina, 2018^[37]^	49/49	SRT + VT	SRT	12	MPT, VHI-T, VHI-E, VHI-F, VHI-P, Jitter, Shimmer, DSI
Ling, 2018^[38]^	13/13	Surgery + VT	Surgery	2	Jitter, Shimmer
Mengyue, 2019^[39]^	100/100	Surgery + VT	Surgery	-	Jitter, Shimmer, DSI
Qiang, 2016^[40]^	82/82	SRT + VT	SRT	-	MPT, VHI-T, Jitter, Shimmer, NHR
Qingqing, 2024^[41]^1	40/41	SRT + VT	SRT	2	Total effective rate, VHI-E, VHI-F, VHI-P
Ronghui, 2023^[42]^	25/25	Surgery + VT	Surgery	4	Total effective rate, VHI-T, VHI-E, VHI-F, VHI-P, G, R, B
Wu, 2020^[43]^	40/40	SRT + VT	SRT	4	MPT, total effective rate, Jitter, Shimmer, DSI
Xiaohong, 2022^[44]^	44/44	SRT + VT	SRT	4	Jitter, Shimmer, VHI-T
Xiaohua, 2021^[45]^	40/40	SRT + VT	SRT	4	MPT, Jitter, Shimmer, G, R, B, DSI
Xiuying, 2017^[46]^	49/49	Surgery + VT	Surgery	4	Jitter, Shimmer, DSI
Yahong, 2019^[47]^	54/54	SRT + VT	SRT	2	MPT, Total effective rate, Jitter, Shimmer, NHR
Yongpeng, 2021^[48]^	50/50	SRT + VT	SRT	12	Jitter, Shimmer, NHR
You, 2017^[49]^	55/41	Surgery + VT	Surgery	12	MPT, VHI-T, VHI-E, VHI-F, VHI-P, Jitter, DSI
Zhaoming, 2017^[50]^	52/52	Surgery + VT	Surgery	12	Total effective rate

B = breathiness, DSI = dysphonia severity index, E = emotional score, F = functional score, G = grade, MPT = maximum phonation time, NHR = noise-harmonic ratio, P = physiological score, R = roughness, SRT = surgery and routine therapy (i.e., routine nursing and conventional treatment), T = total, VHI = voice handicap index, VT = voice training.

### 3.3. Pooled analyses

**Figure 2.**
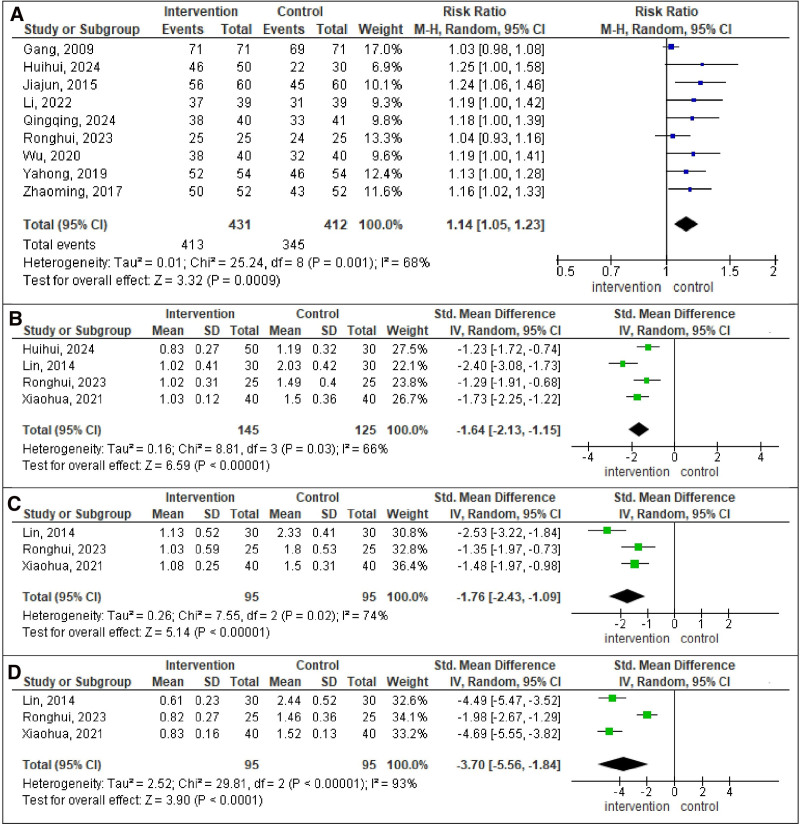
Forest plot analysis. (A) Total effective rate. (B) Grade. (C) Roughness. (D) Breathiness. Each plot displays individual study results as squares, with the size indicating the study’s weight and horizontal lines representing CIs. The vertical line marks no effect. At the bottom of each plot, a diamond represents the pooled result of all studies, with its width showing the CI. If the diamond does not cross the vertical line, it indicates a statistically significant result. CI = confidence interval.

Figure 2 presents 4 forest plots, each analyzing different outcomes in intervention group compared to control group. The pooled RR for total effective rate was 1.14 (95% CI: 1.05, 1.23; *P* = .0009; *I*^2^ = 68%). The pooled SMD for G was −1.64 (95% CI: −2.13, −1.15; *P* < .00001; *I*^2^ = 66%), for R was −1.76 (95% CI: −2.43, −1.09; *P* < .00001; *I*^2^ = 74%), and for B was −3.70 (95% CI: −5.56, −1.84; *P* < .0001; *I*^2^ = 93%). The results showed a 14% improvement in the intervention group compared to the control group. In addition, there was a significant reduction of G, R, and B in the intervention group compared to the control group.

**Figure 3.**
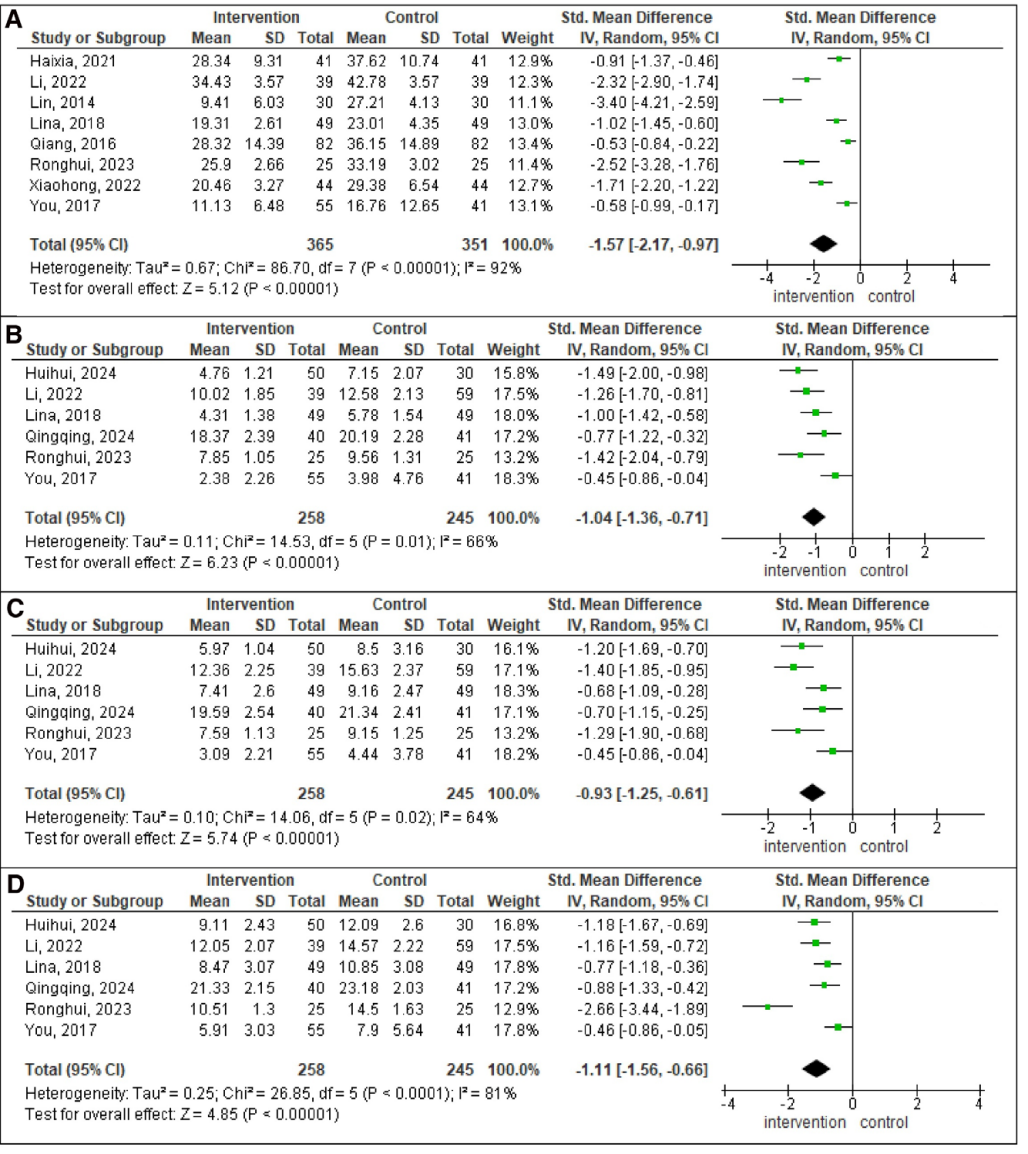
Forest plot analysis. (A) VHI-T. (B) VHI-E. (C) VHI-F. (D) VHI-P. Each plot displays individual study results as squares, with the size indicating the study’s weight and horizontal lines representing CIs. The vertical line marks no effect. At the bottom of each plot, a diamond represents the pooled result of all studies, with its width showing the CI. If the diamond does not cross the vertical line, it indicates a statistically significant result. E = emotional score, F = functional score, P = physiological score, T = total, VHI = voice handicap index.

Figure 3 presents 4 forest plots, each analyzing different outcomes in intervention group compared to control group. The pooled SMD for VHI-T was −1.57 (95% CI: −2.17, −0.97; *P* < .00001; *I*^2^ = 92%), for VHI-E was −1.04 (95% CI: −1.36, −0.71; *P* < .00001; *I*^2^ = 66%), for VHI-F was −0.93 (95% CI: −1.25, −0.61; *P* < .00001; *I*^2^ = 64%), and for VHI-P was −1.11 (95% CI: −1.56, −0.66; *P* < .00001; *I*^2^ = 81%). There was a significant reduction of VHI-T, VHI-E, VHI-F, and VHI-P in the intervention group compared to the control group.

**Figure 4.**
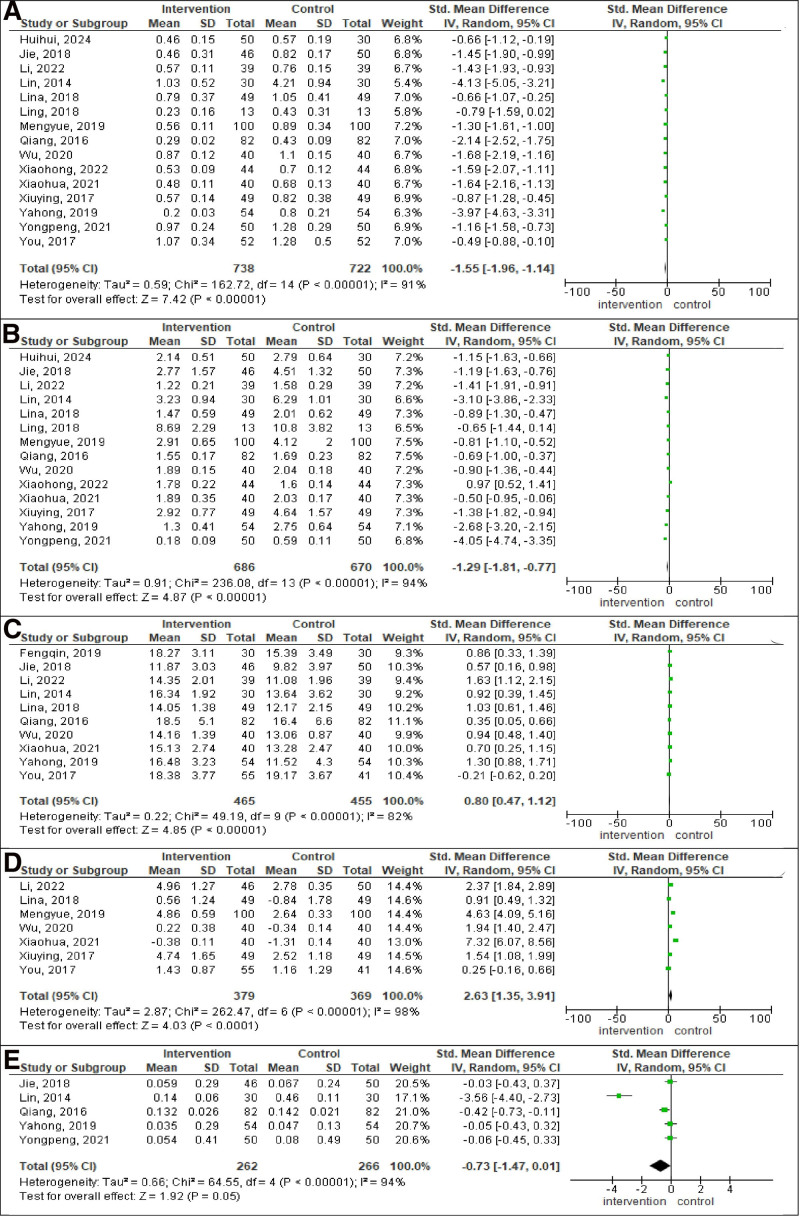
Forest plot analysis. (A) Jitter. (B) Shimmer. (C) MPT. (D) DSI. (E) NHR. Each plot displays individual study results as squares, with the size indicating the study’s weight and horizontal lines representing CIs. The vertical line marks no effect. At the bottom of each plot, a diamond represents the pooled result of all studies, with its width showing the CI. If the diamond does not cross the vertical line, it indicates a statistically significant result. CI = confidence interval, DSI = dysphonia severity index, MPT = maximum phonation time, NHR = noise-harmonic ratio.

Figure 4 presents 5 forest plots, each analyzing different outcomes in intervention group compared to control group. The pooled SMD for jitter was −1.55 (95% CI: −1.96, −1.14; *P* < .00001; *I*^2^ = 91%), for shimmer was −1.29 (95% CI: −1.81, −0.77; *P* < .00001; *I*^2^ = 94%), for MPT was 0.80 (95% CI: 0.47, 1.12; *P* < .00001; *I*^2^ = 82%), for DSI was 2.63 (95% CI: 1.35, 3.91; *P* < .0001; *I*^2^ = 98%), for NHR was −0.73 (95% CI: −1.47, −0.01; *P* = .05; *I*^2^ = 94%). There was a significant reduction of jitter and shimmer and a significant increase of MPT and DSI in the intervention group compared to the control group. But there was no significant difference between 2 groups for NHR.

### 3.4. Sensitivity analysis

Both sensitivity analyses (one-study-removed and cumulative analyses) didn’t change the pooled results and therefore, the results were stable.

### 3.5. Risk of bias

**Figure 5.**
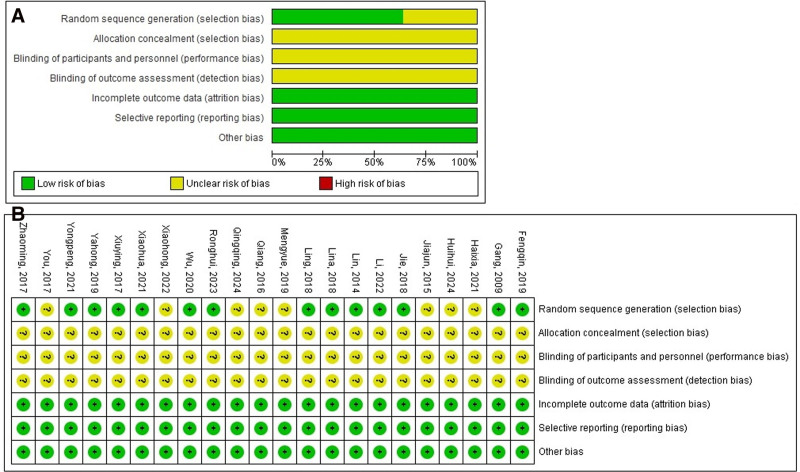
Bias risk assessment. (A) Risk of bias summary. (B) Risk of bias graph.

Eight studies didn’t report random sequence generation, all studies didn’t report allocation concealment, and performance, and detection biases (Fig. 5).

### 3.6. TSA

Figures S1–S9 (Supplemental Digital Content, https://links.lww.com/MD/P820) show TSA plots. The results indicated that there is sufficient evidence to conclude a significant effect for the VHI-T, jitter, shimmer, MPT, and DSI. This is because the Z-curve (cumulative Z-score) crossed the RIS line for these outcomes, suggesting that the RIS has been reached and the findings are likely conclusive. However, for total effective rate, VHI-E, VHI-F, and VHI-P, the Z-curve did not cross the RIS line. This suggests that the current information size (i.e., the number of participants in the studies analyzed) may not be sufficient to draw a definitive conclusion and more studies may be needed to confirm the effect of VT on these outcomes.

### 3.7. Publication bias

Figures S1–S13 (Supplemental Digital Content, https://links.lww.com/MD/P820) show the funnel plots for all outcomes. Egger and Begg tests revealed publication bias for DSI (*P*-values of .03373 and .01067), jitter (*P*-values of .06010 and .02595), shimmer (*P*-values of .06591 and .04281), VHI-P (*P*-values of .00627 and .01460), and VHI-T (*P*-values of .00053 and .00299). Just Begg test reveled publication bias for VHI-E (*P*-value of .09087) and VHI-F (*P*-value of .09087) and Egger test for NHR (*P*-value of .08799).

## 4. Discussion

The total effective rate was improved by 14% (RR = 1.14), with notable reductions in G, R, and B (SMDs: −1.64, −1.76, −3.70). VHI-T, VHI-E, VHI-F, and VHI-P also showed significant reductions (SMDs: −1.57, −1.04, −0.93, −1.11). Additionally, jitter and shimmer decreased (SMDs: −1.55, −1.29), while MPT and DSI increased (SMDs: 0.80, 2.63), with no significant change in NHR. Risk of bias was noted due to inadequate reporting on random sequence generation and allocation concealment. TSA plots confirmed sufficient evidence for VHI-T, jitter, shimmer, MPT, and DSI, while funnel plots indicated publication bias for several outcomes.

Recent in-depth research on the pathophysiology and pathogenesis of BVLs has spotlighted the effectiveness of VT for treating BVLs.^[51]^ Most studies have focused on voice microsurgery, with related reports affirming its efficacy for BVLs.^[52–54]^ Researchers suggest that smaller BVLs are linked to better outcomes with VT and health education.^[55]^ One study^[56]^ systematically reviewed the acoustic, aerodynamic, and self-evaluated changes in vocal disorder for BVL patients post-voice microsurgery, finding VT improved patients’ self-evaluations. Another study^[57]^ found that acoustic parameters of BVL patients improved after phonomicrosurgery and VT, approaching control group values. This provides robust clinical evidence and guidance for applying voice microsurgery and VT effectively.

VT helps patients better understand their voice issues, enhance vocal comfort with treatment, and set realistic treatment expectations, thereby improving their self-perceived voice handicap.^[49]^ By recognizing voice problems more clearly and improving vocal comfort, VT aims to establish and reestablish physiological balance among the vocal organs and correct the erroneous conditioned reflex caused by vocal cord lesions. Changing the vocal environment and condition helps eliminate the control of the faulty reflex, alter vocal habits, and establish a new reflex for normal phonation function.^[14,15]^

To further substantiate these findings, it is essential to conduct well-structured, large-scale randomized controlled trials to minimize biases and strengthen the evidence base. Future research should focus on the long-term effects of VT post-microsurgery, exploring the underlying mechanisms through advanced imaging and acoustic analysis technologies. Additionally, it would be beneficial to investigate the efficacy of personalized VT protocols tailored to individual patient profiles. A comprehensive approach integrating patient education, regular follow-ups, and multidisciplinary collaboration could optimize outcomes and enhance the quality of life for patients with BVLs. By addressing these areas, we can significantly advance our understanding and management of BVLs, ultimately improving patient care and therapeutic strategies.

### 4.1. Limitations

1. Risk of bias: The lack of reporting on random sequence generation in several studies and allocation concealment in all studies introduces potential biases that could affect the reliability of the results.
2. Heterogeneity: High *I*² values in some outcomes indicate substantial heterogeneity, suggesting variability in study designs, populations, or interventions that could influence the pooled estimates.
3. Publication bias: Egger and Begg tests revealed publication bias for several outcomes, which may overestimate the intervention’s effectiveness.
4. Sample size: For some outcomes, the current information size may not be sufficient to draw definitive conclusions, indicating a need for more extensive studies.
5. Generalizability: The findings may not be generalizable to all populations or settings, as the included studies may have specific inclusion criteria or demographic characteristics.

## 5. Conclusions

The intervention group demonstrated significant improvements across various outcomes compared to the control group, including a 14% increase in total effective rate and reductions in G, R, B, VHI-T, VHI-E, VHI-F, VHI-P, jitter, and shimmer. Additionally, there were significant increases in MPT and DSI, with no significant change in NHR. However, the risk of bias due to inadequate reporting on random sequence generation and allocation concealment should be considered.

These findings suggest that the intervention is effective in improving clinical outcomes, particularly in reducing symptoms and enhancing functional measures. The significant improvements in VHI and voice parameters indicate potential benefits for patients with voice disorders, contributing to better quality of life and clinical management.

Future research should focus on addressing the identified biases by ensuring rigorous study designs with proper randomization and allocation concealment. Additionally, further studies with larger sample sizes are needed to confirm these findings and explore the long-term effects of the intervention. Investigating the underlying mechanisms of action and potential side effects will also be crucial for optimizing treatment protocols and improving patient outcomes.

## Acknowledgments

The authors would like to thank their families and the hospital leadership for their support and assistance in completing this study.

## Author contributions

**Conceptualization:** Xiaolan Zhang, Chaobing Liu.

**Data curation:** Xiaolan Zhang, Chaobing Liu.

**Formal analysis:** Chaobing Liu.

**Funding acquisition:** Zhitao Fan.

**Investigation:** Xiaolan Zhang.

**Methodology:** Chaobing Liu, Man Liu.

**Project administration:** Xiaolan Zhang.

**Resources:** Chaobing Liu, Zhenhua Qiao.

**Software:** Zhitao Fan, Man Liu.

**Supervision:** Xiaolan Zhang.

**Validation:** Zhenhua Qiao.

**Visualization:** Zhitao Fan.

**Writing – review & editing:** Xiaolan Zhang, Chaobing Liu, Zhitao Fan, Zhenhua Qiao, Man Liu.

**Writing – original draft:** Zhitao Fan.

## Supplementary Material


