# Supplementary material for: Efficacy of voice training on benign vocal cord lesions after surgery: A systematic review, meta-analysis, and trial sequential analysis of randomized clinical trials
Source: Medicine (Baltimore). 2025 Aug 29;104(35):e44024. doi: 10.1097/MD.0000000000044024 (PMC12401348; doi:10.1097/MD.0000000000044024)
Supplement: Supplementary file 1 [file medi-104-e44024-s001.pdf]

Supplementary Digital Content 1

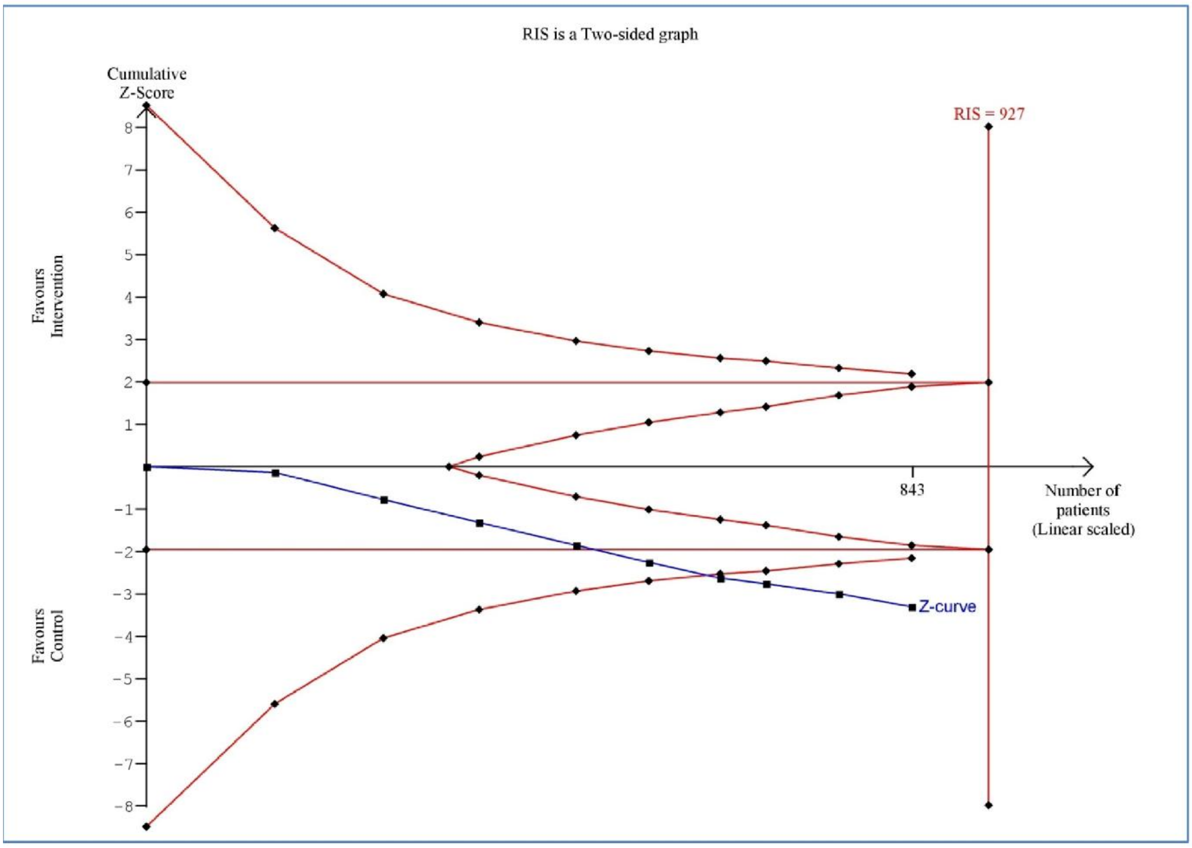

Figure S1-1: Funnel plot of total effective rate

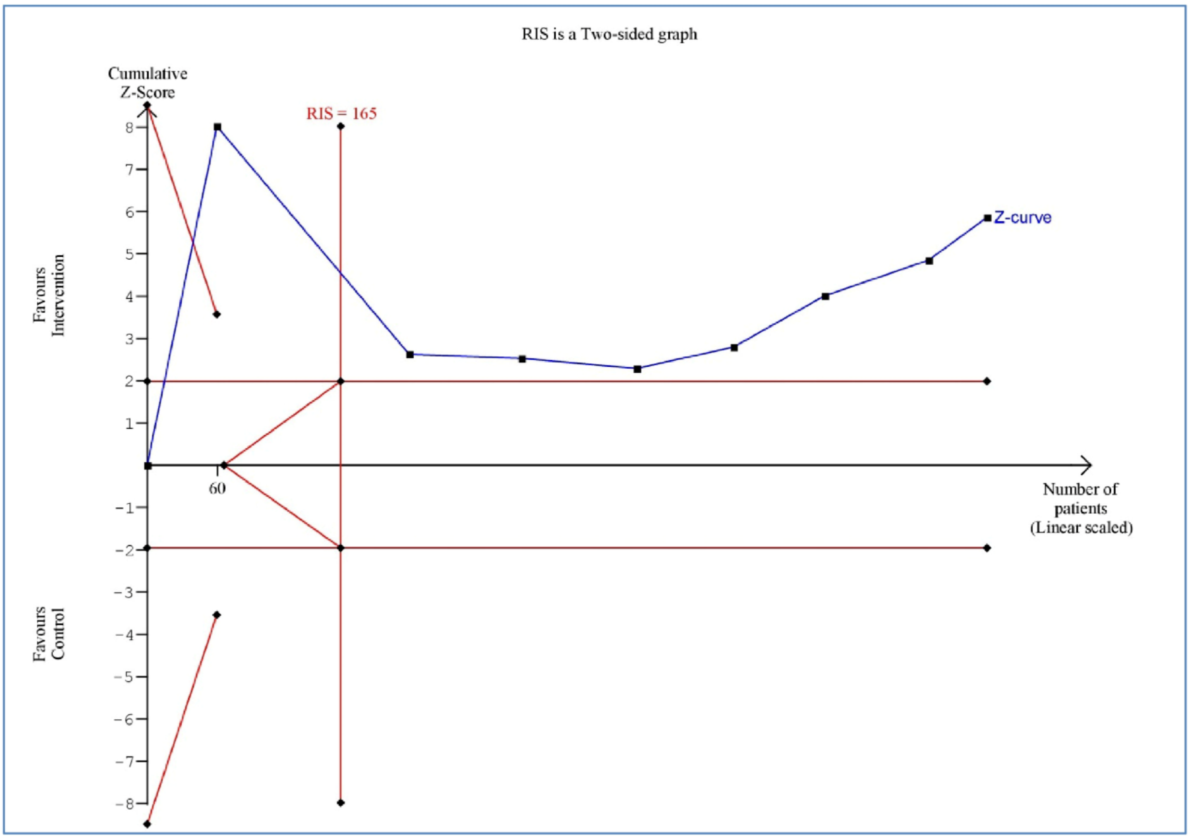

Figure S1-2: Funnel plot of VHI-T

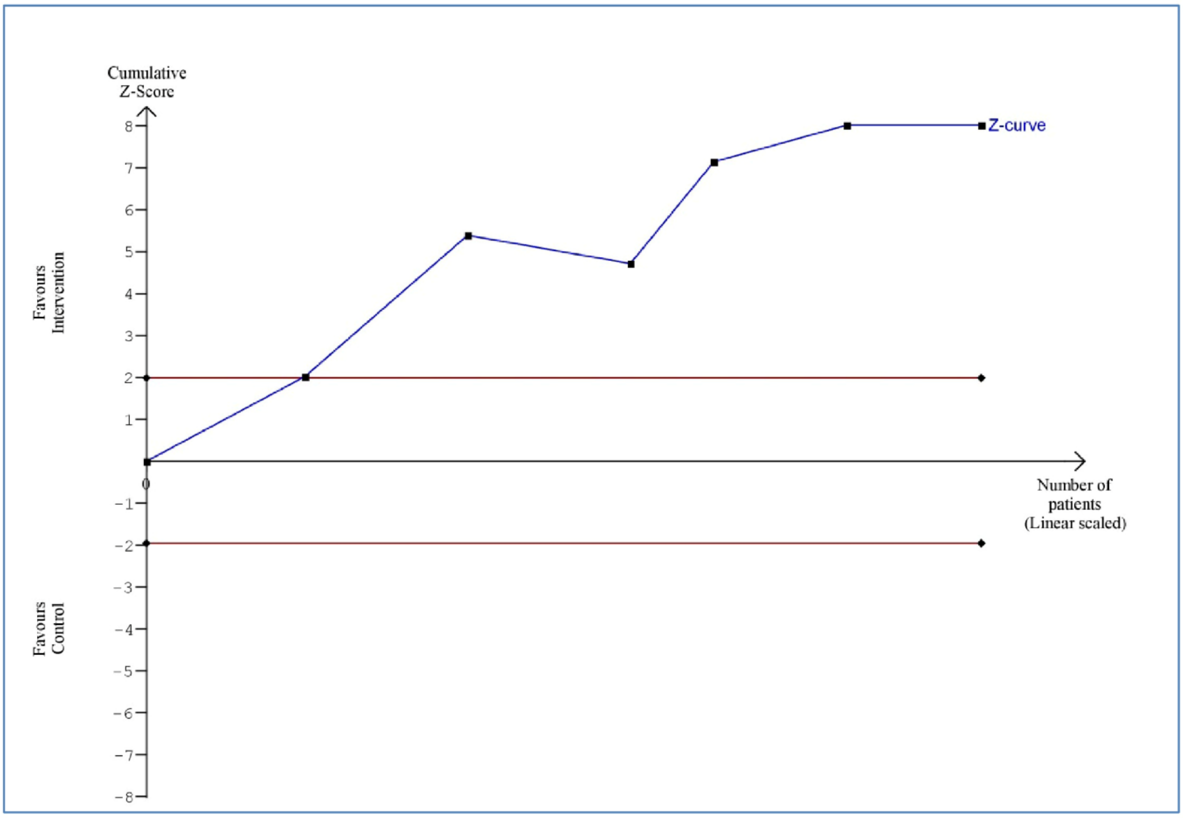

Figure S1-3: Funnel plot of VHI-E

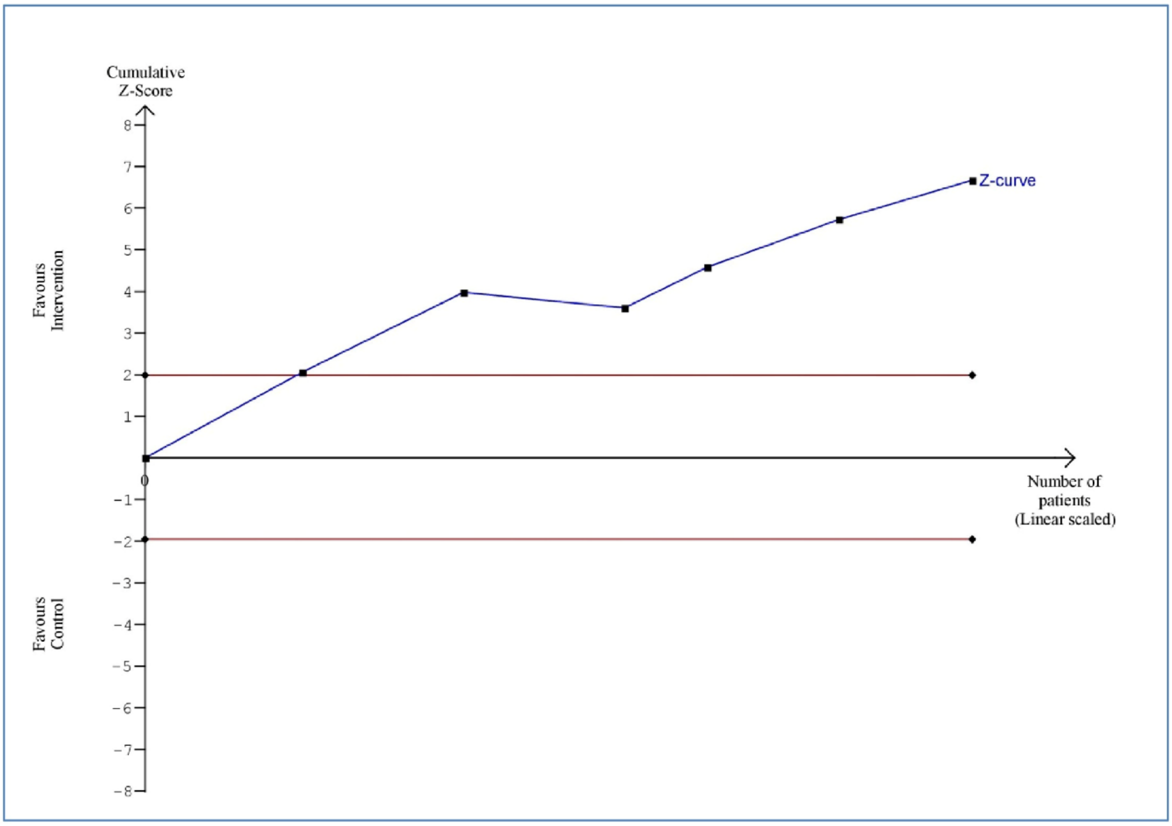

Figure S1-4: Funnel plot of VHI-F

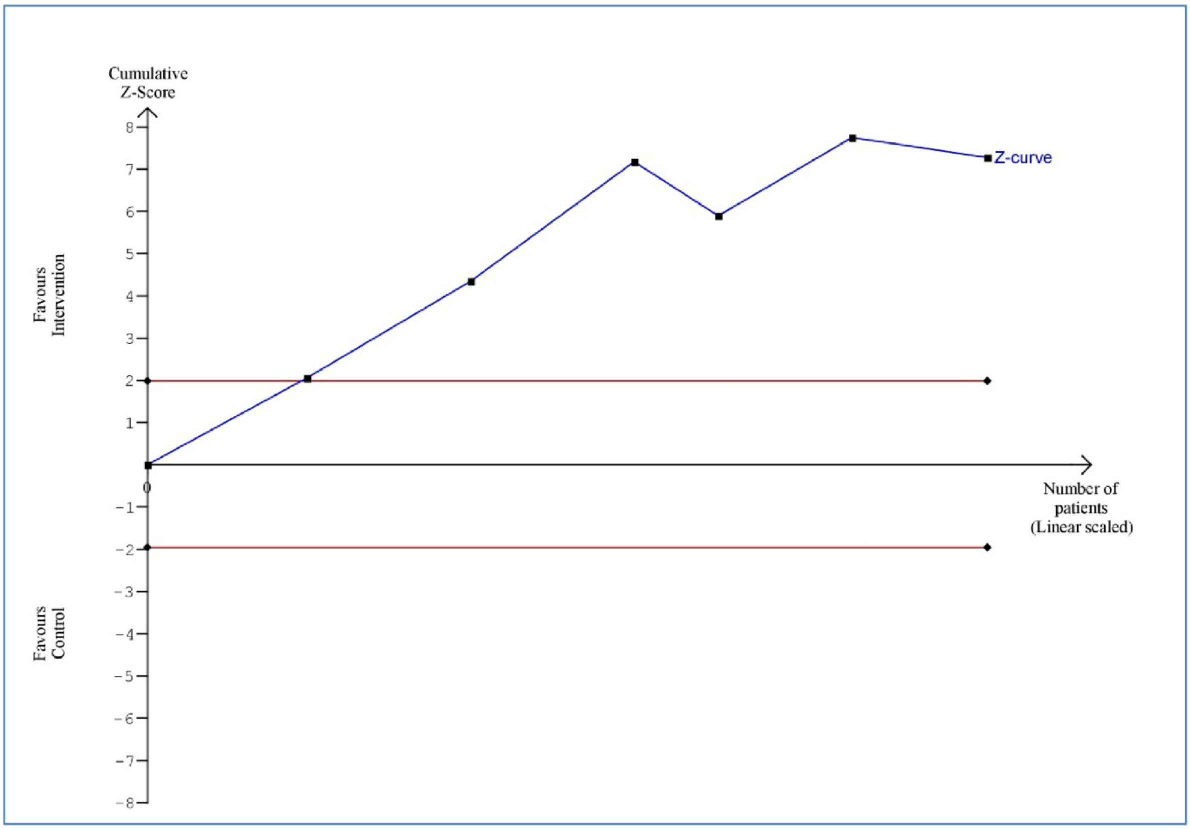

Figure S1-5: Funnel plot of VHI-P

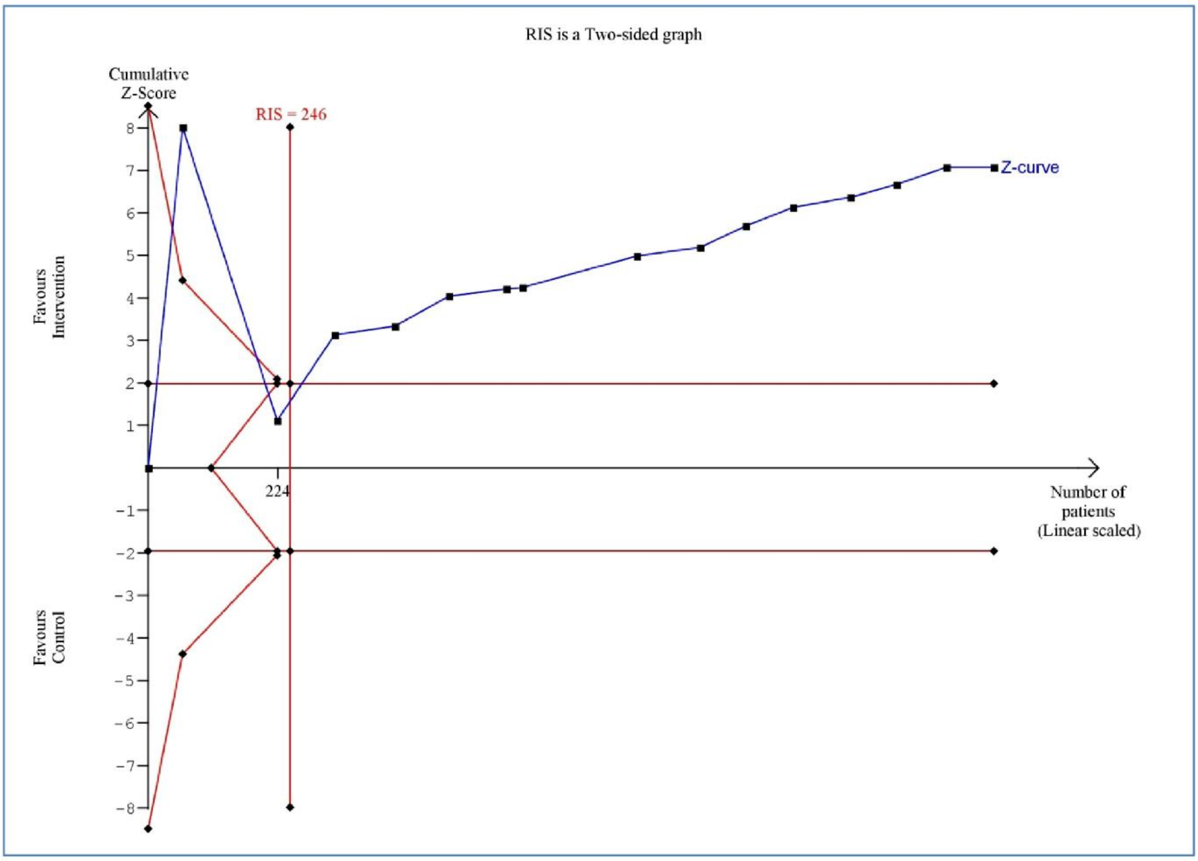

Figure S1-6: Funnel plot of jitter

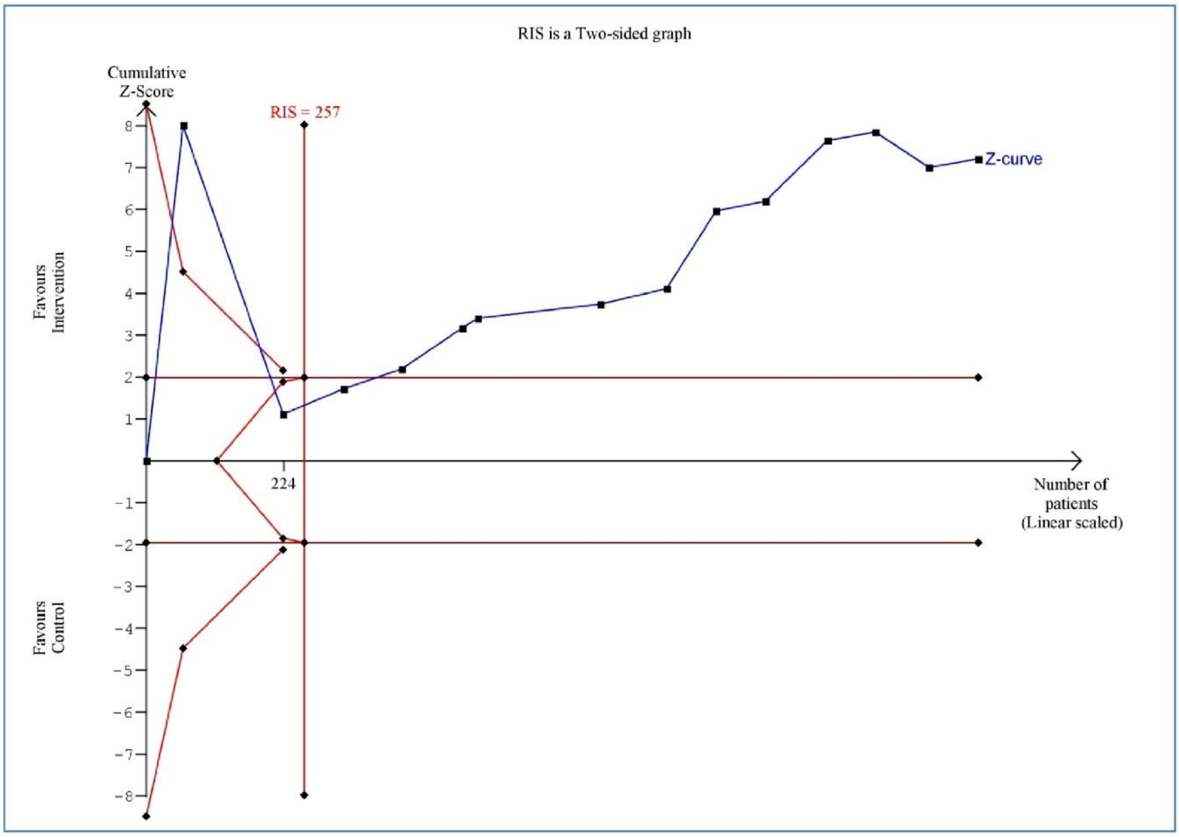

Figure S1-7: Funnel plot of shimmer

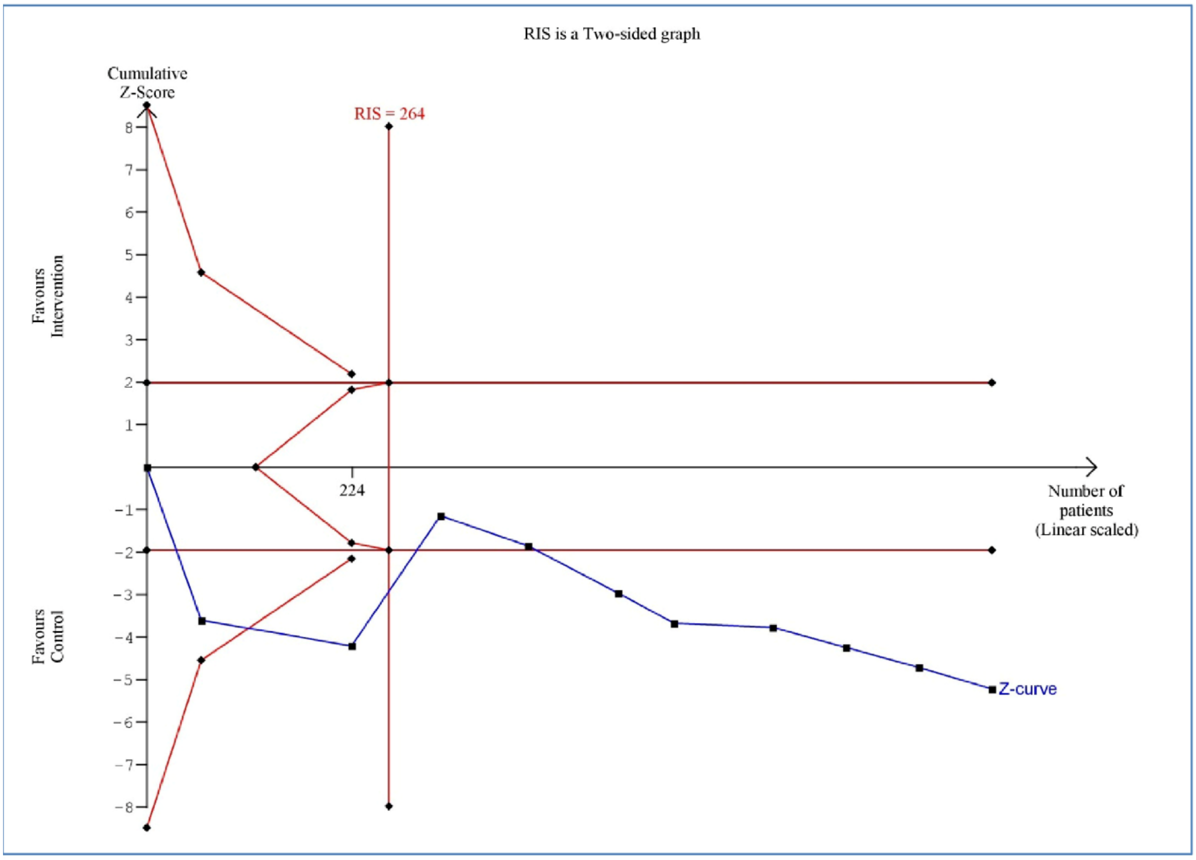

Figure S1-8: Funnel plot of MPT

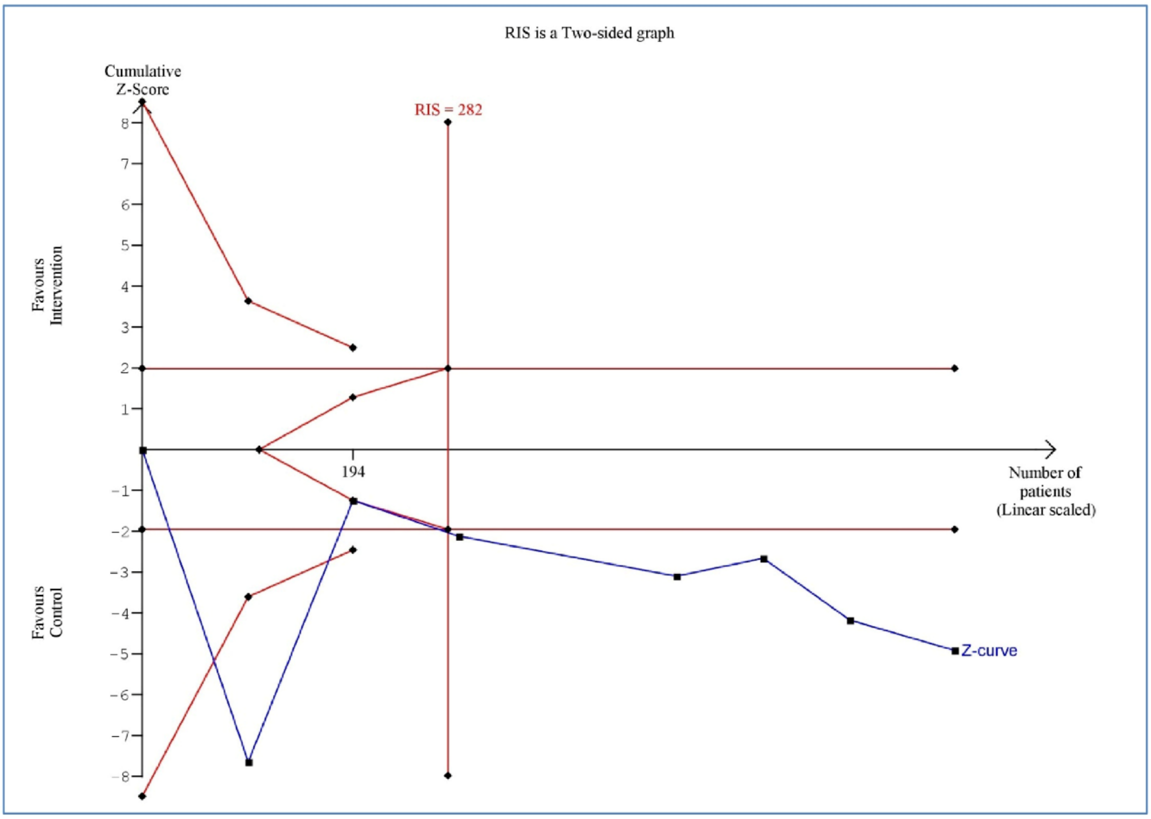

Figure S1-9: Funnel plot of DSI

Supplementary Digital Content 2

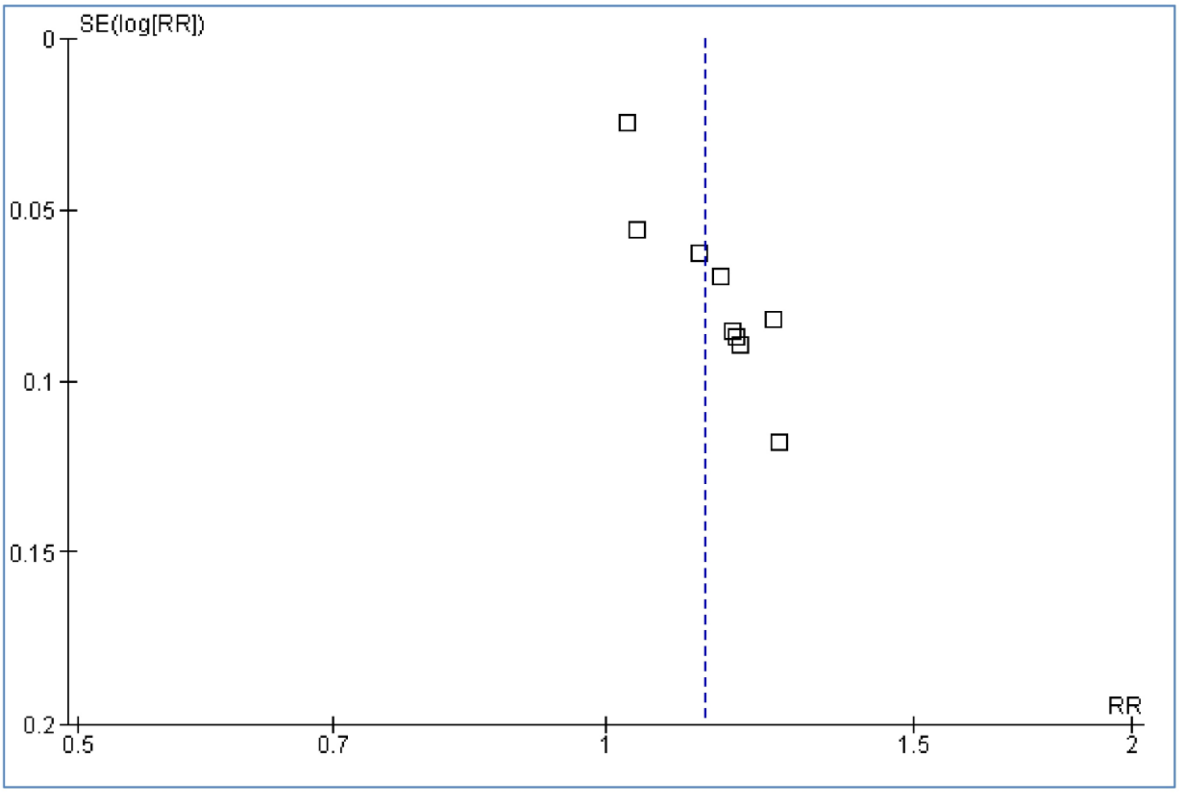

Figure S2-1: Funnel plot of total effective rate

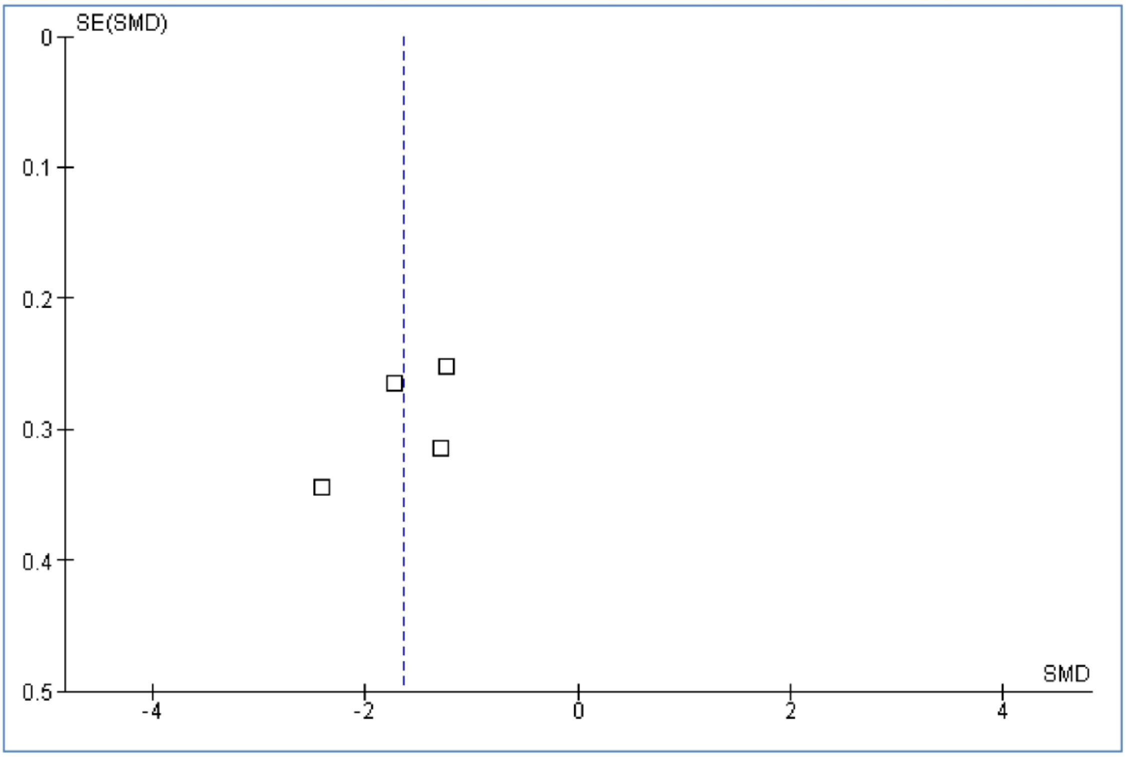

Figure S2-2: Funnel plot of grade

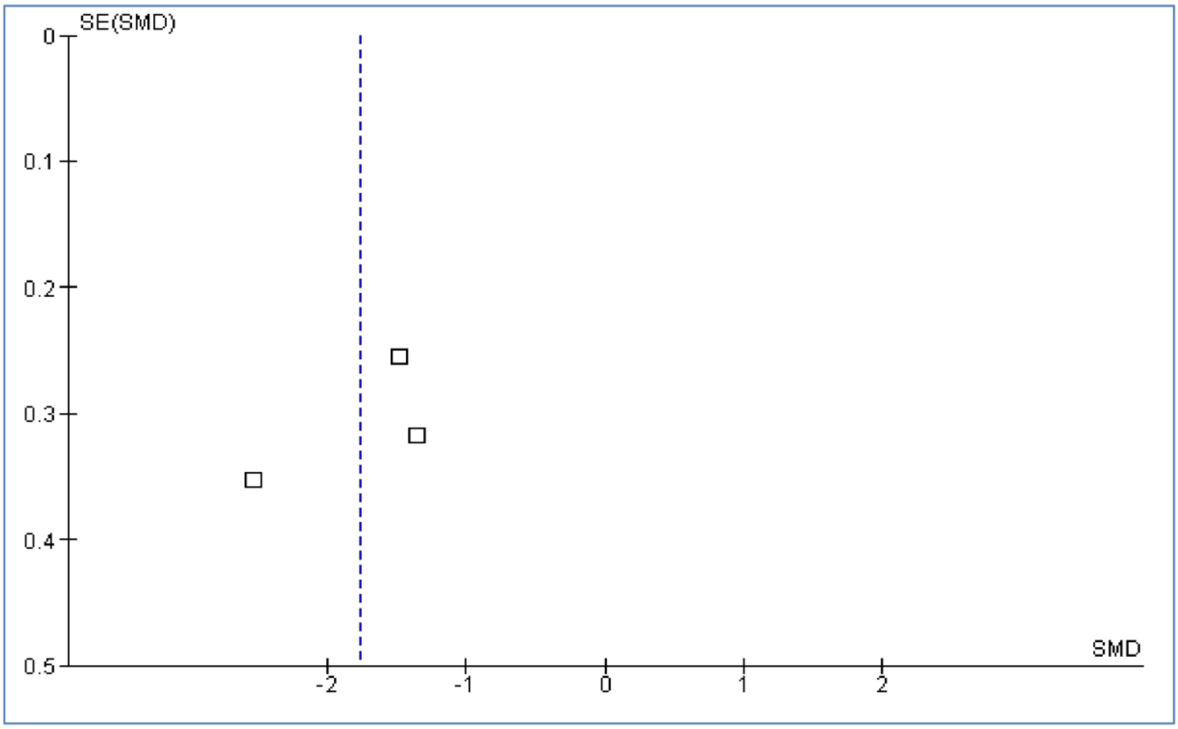

Figure S2-3: Funnel plot of roughness

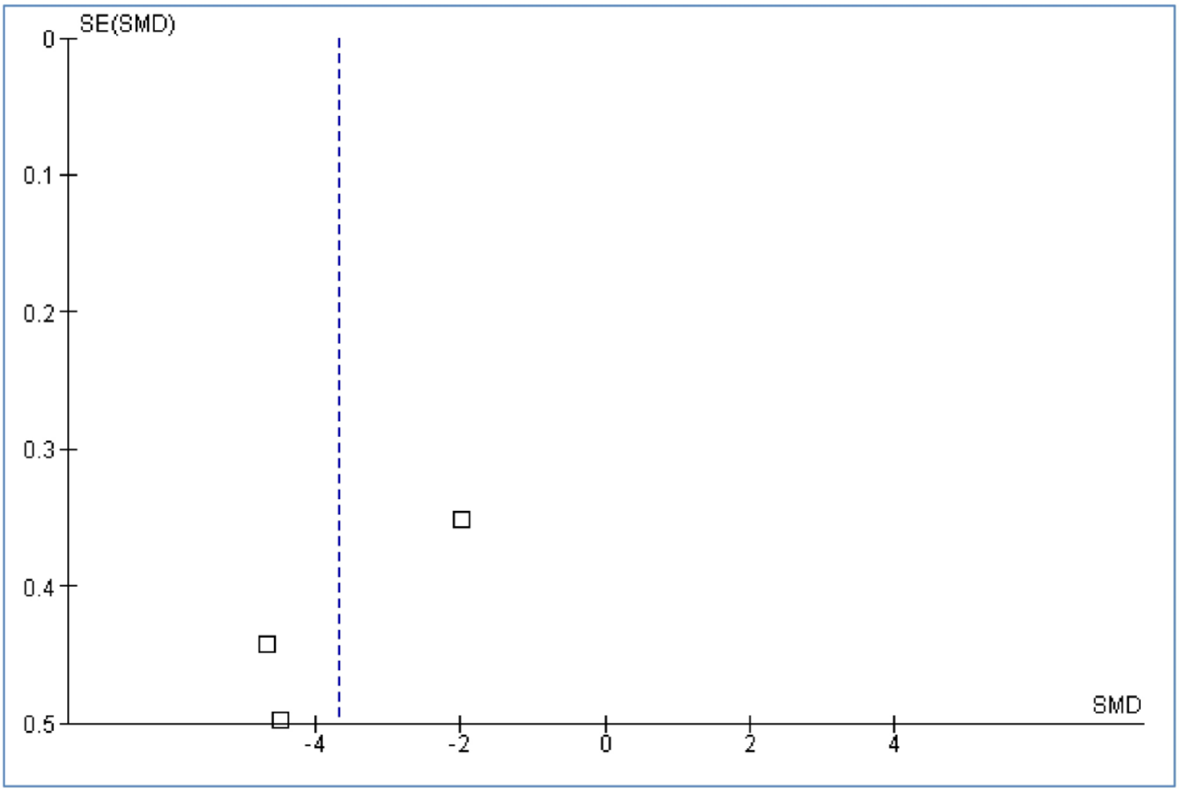

Figure S2-4: Funnel plot of breathiness

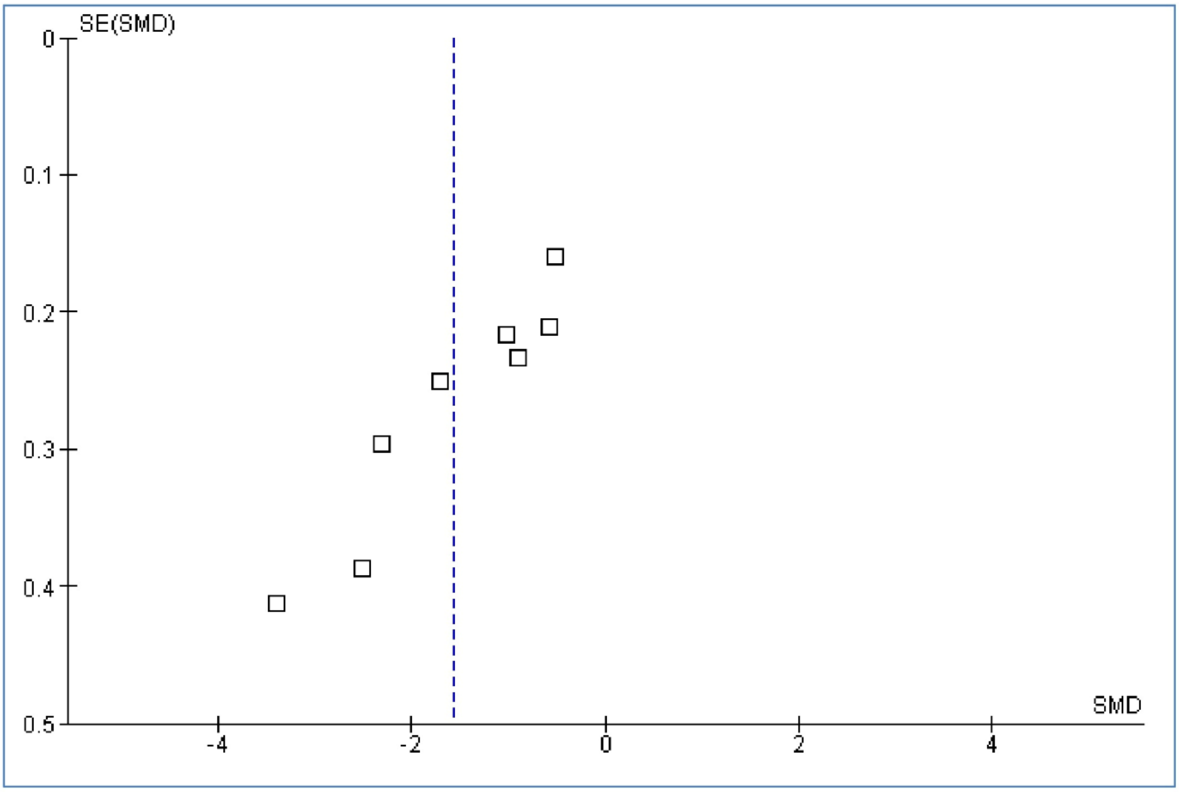

Figure S2-5: Funnel plot of VHI-T

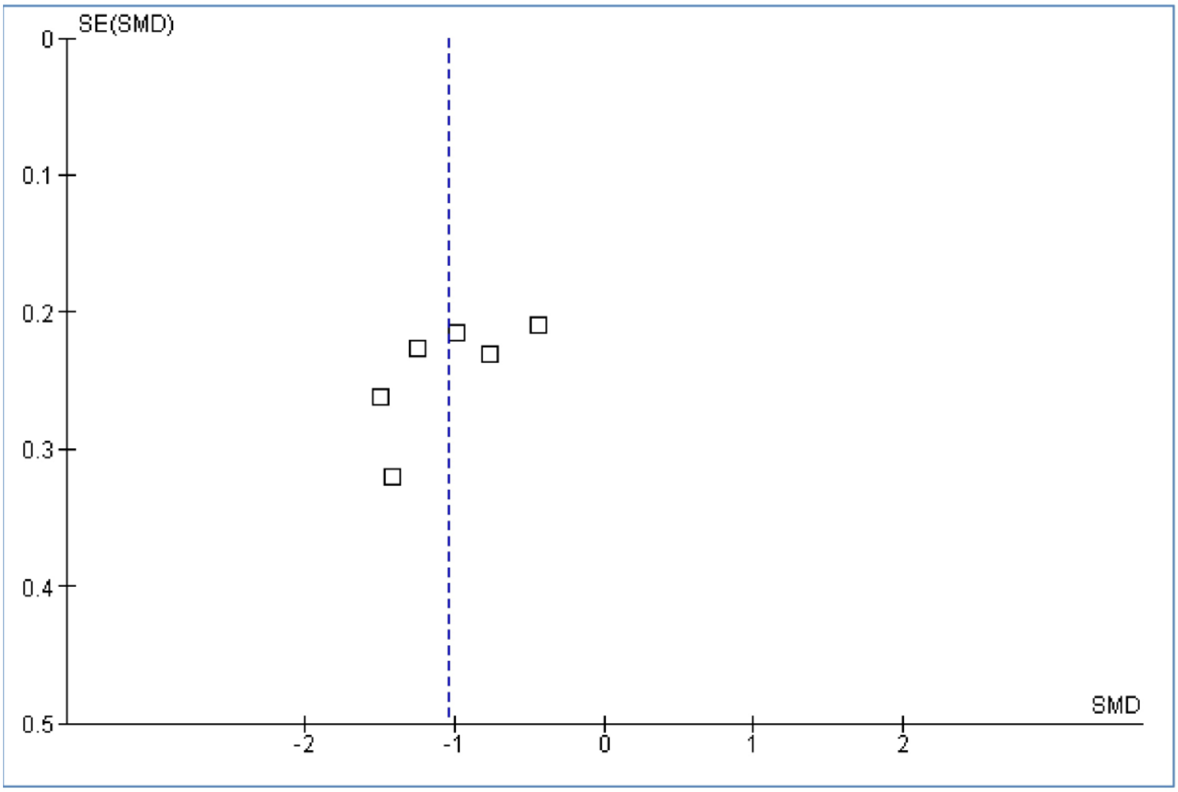

Figure S2-6: Funnel plot of VHI-E

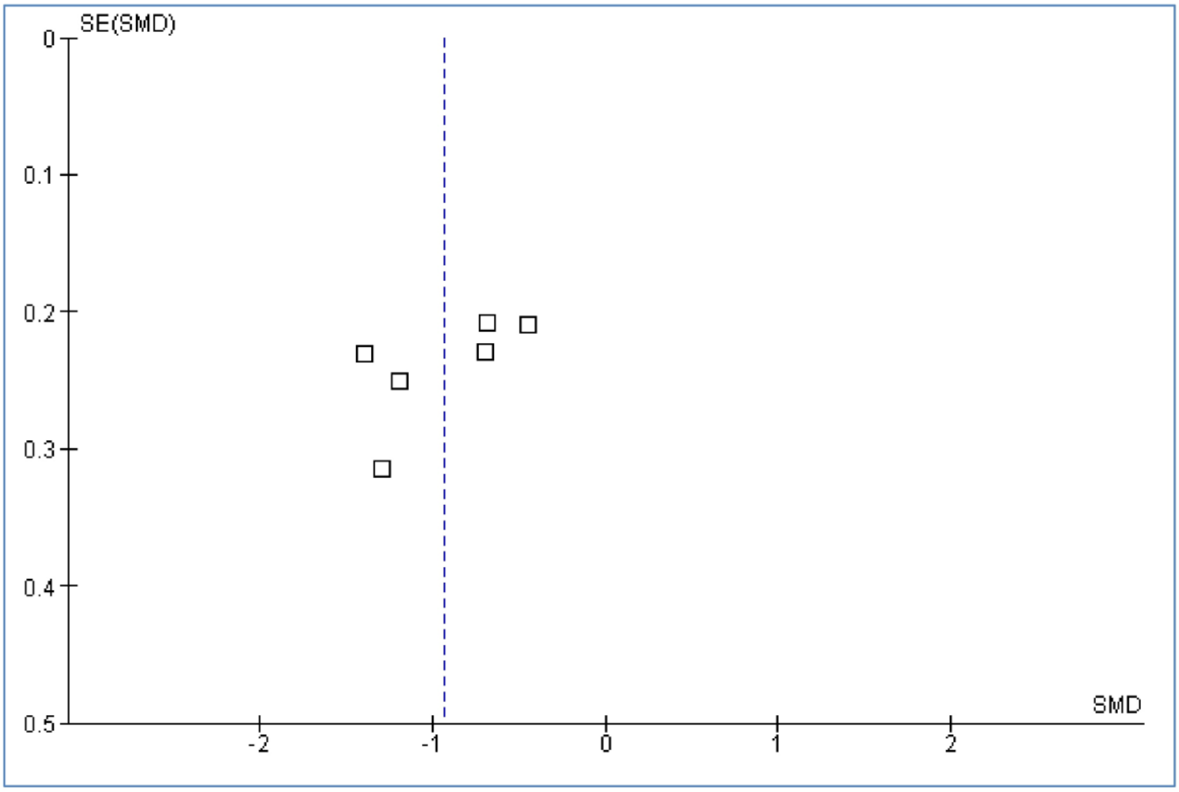

Figure S2-7: Funnel plot of VHI-F

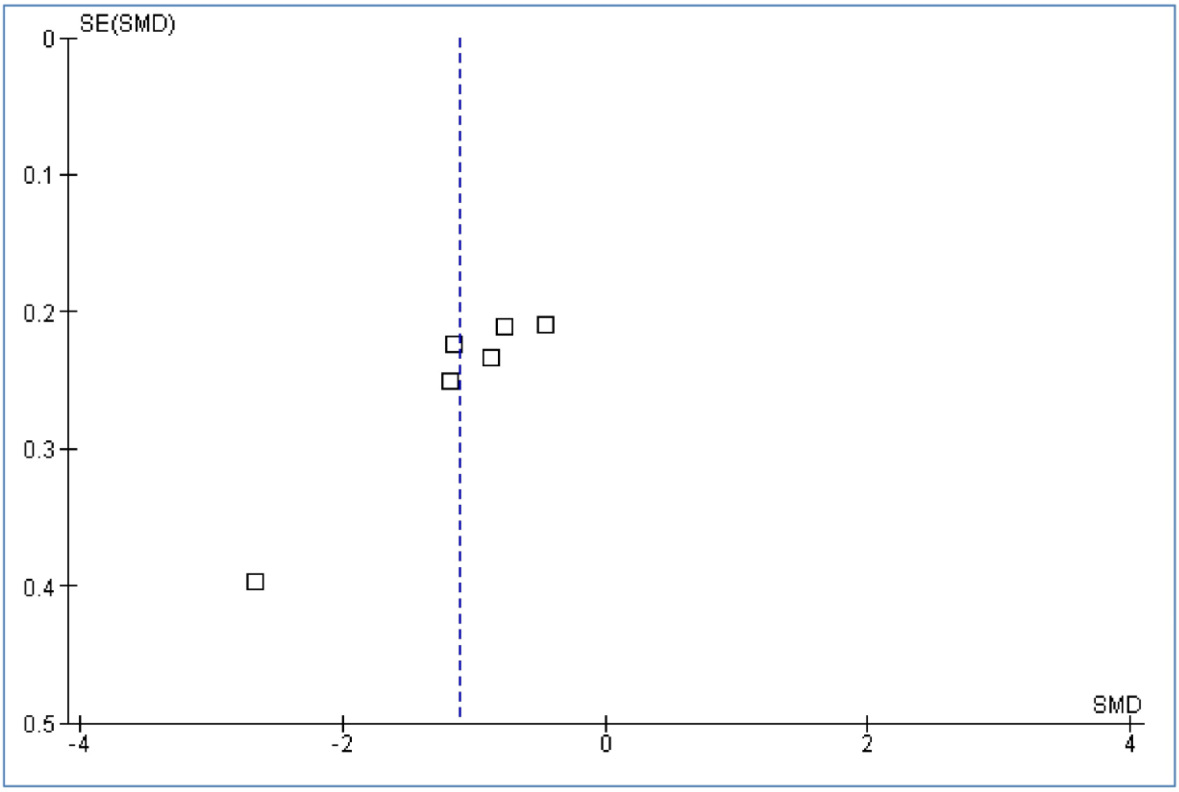

Figure S2-8: Funnel plot of VHI-P

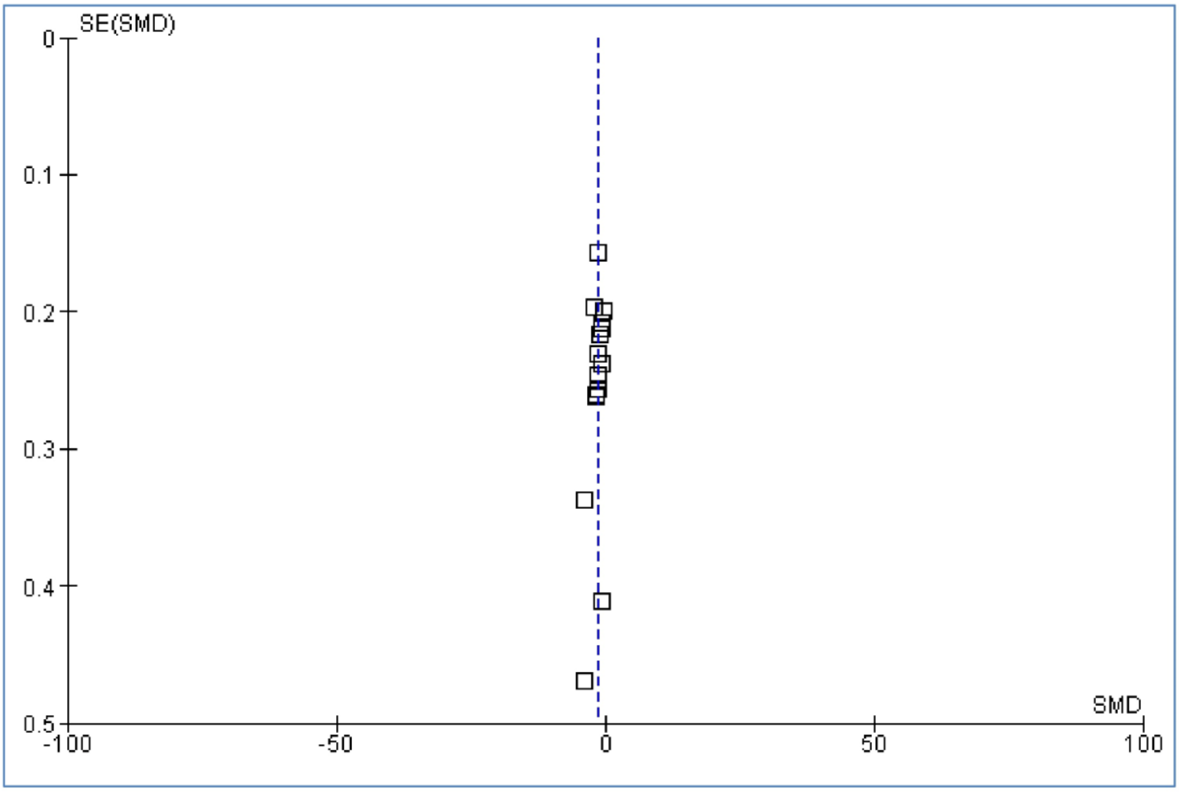

Figure S2-9: Funnel plot of jitter

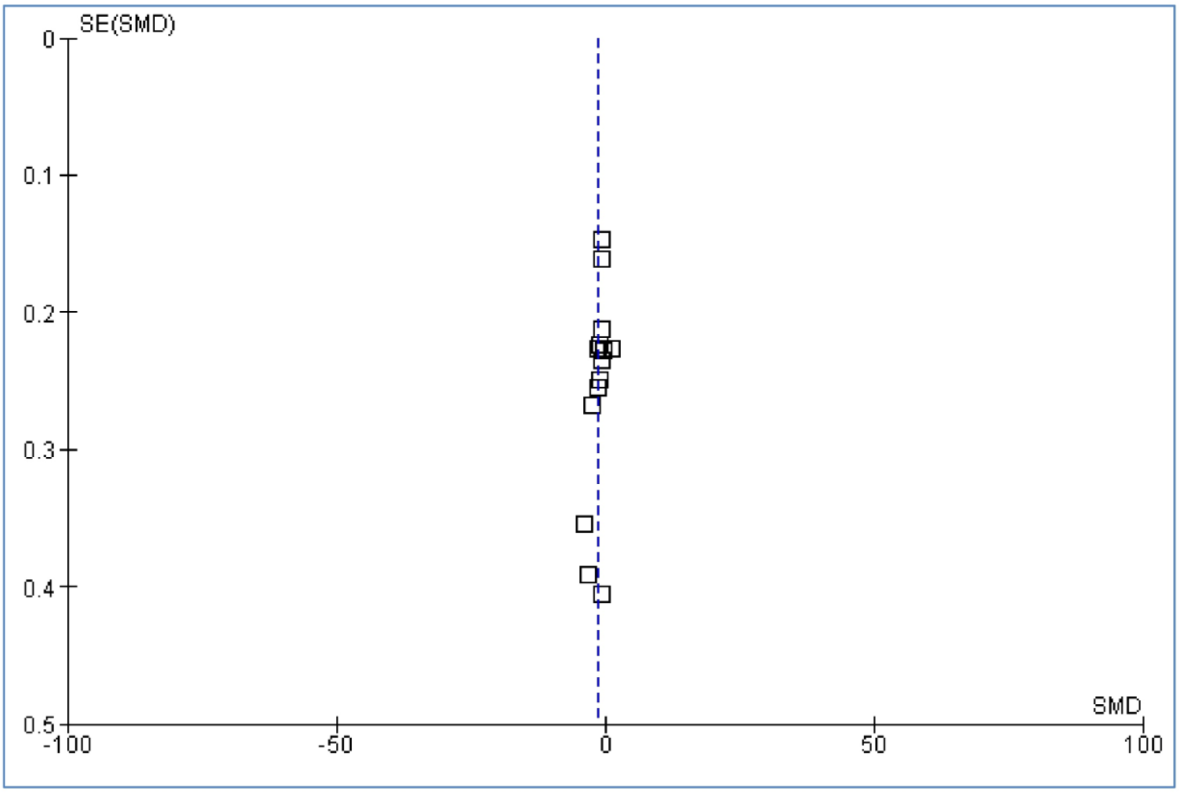

Figure S2-10: Funnel plot of shimmer

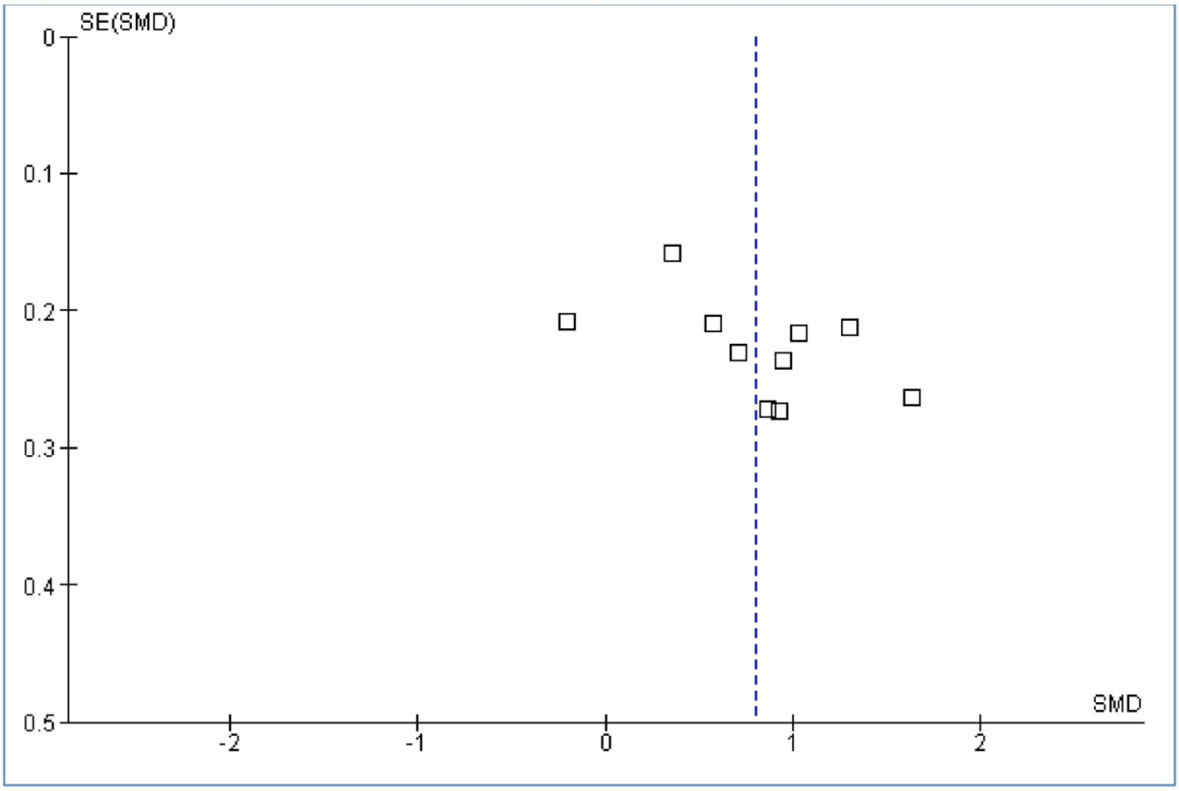

Figure S2-11: Funnel plot of MPT

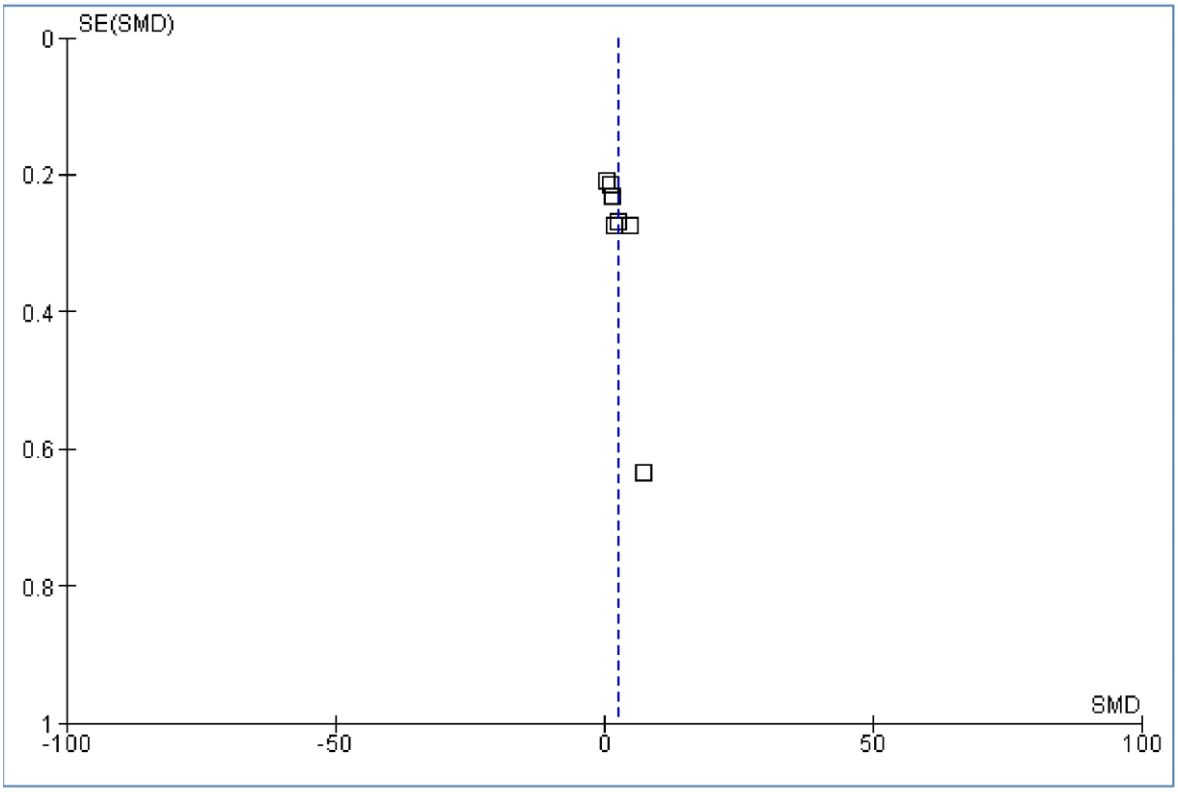

Figure S2-12: Funnel plot of DSI

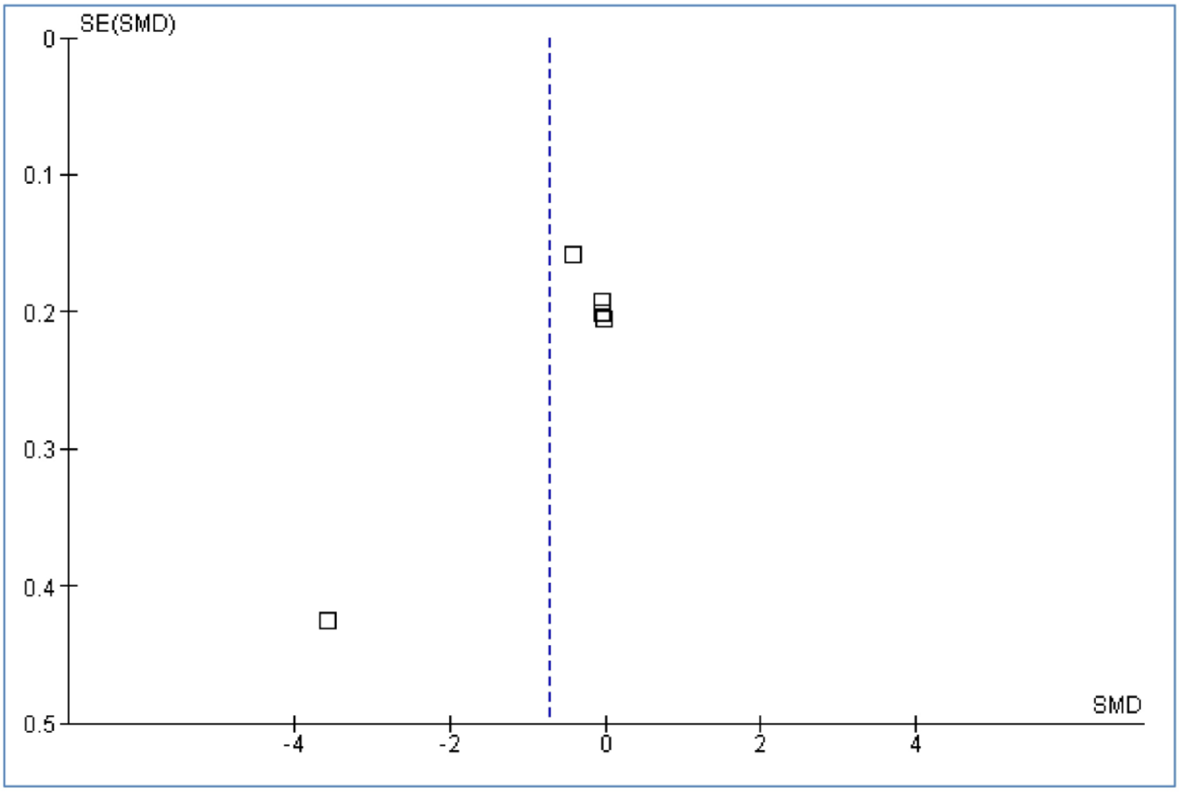

Figure S2-13: Funnel plot of NHR
